# Neurotransmitter System-Targeting Drugs Antagonize Growth of the Q Fever Agent, Coxiella burnetii, in Human Cells

**DOI:** 10.1128/mSphere.00442-21

**Published:** 2021-07-07

**Authors:** Marissa S. Fullerton, Punsiri M. Colonne, Amanda L. Dragan, Katelynn R. Brann, Richard C. Kurten, Daniel E. Voth

**Affiliations:** a Department of Microbiology and Immunology, University of Arkansas for Medical Sciences, Little Rock, Arkansas, USA; b Department of Physiology and Biophysics, University of Arkansas for Medical Sciences, Little Rock, Arkansas, USA; c Arkansas Children’s Research Institute, Little Rock, Arkansas, USA; University of Florida

**Keywords:** *Coxiella burnetii*, antibiotic resistance, intracellular pathogen, macrophage, neurotransmitter systems

## Abstract

Coxiella burnetii is a highly infectious, intracellular, Gram-negative bacterial pathogen that causes human Q fever, an acute flu-like illness that can progress to chronic endocarditis. C. burnetii is transmitted to humans via aerosols and has long been considered a potential biological warfare agent. Although antibiotics, such as doxycycline, effectively treat acute Q fever, a recently identified antibiotic-resistant strain demonstrates the ability of C. burnetii to resist traditional antimicrobials, and chronic disease is extremely difficult to treat with current options. These findings highlight the need for new Q fever therapeutics, and repurposed drugs that target eukaryotic functions to prevent bacterial replication are of increasing interest in infectious disease. To identify this class of anti-C. burnetii therapeutics, we screened a library of 727 FDA-approved or late-stage clinical trial compounds using a human macrophage-like cell model of infection. Eighty-eight compounds inhibited bacterial replication, including known antibiotics, antipsychotic or antidepressant treatments, antihistamines, and several additional compounds used to treat a variety of conditions. The majority of identified anti-C. burnetii compounds target host neurotransmitter system components. Serotoninergic, dopaminergic, and adrenergic components are among the most highly represented targets and potentially regulate macrophage activation, cytokine production, and autophagy. Overall, our screen identified multiple host-directed compounds that can be pursued for potential use as anti-C. burnetii drugs.

**IMPORTANCE**Coxiella burnetii causes the debilitating disease Q fever in humans. This infection is difficult to treat with current antibiotics and can progress to long-term, potentially fatal infection in immunocompromised individuals or when treatment is delayed. Here, we identified many new potential treatment options in the form of drugs that are either FDA approved or have been used in late-stage clinical trials and target human neurotransmitter systems. These compounds are poised for future characterization as nontraditional anti-C. burnetii therapies.

## INTRODUCTION

Bacterial resistance to antibiotics is increasingly widespread, and pathogens that exploit eukaryotic cells for intracellular growth are no exception. The host cell cytosol is a complex environment in which to introduce traditional antibiotics, and combination therapy is often required to eradicate bacteria growing within membrane-bound vacuoles. The causative agent of pulmonary human Q fever, Coxiella burnetii, infects alveolar macrophages and grows within a host-derived membranous compartment, termed the parasitophorous vacuole (PV), that is required for disease progression (1–5). The PV fuses with numerous host compartments, including autophagosomes and lysosomes, resulting in an acidic, degradative vacuole that is not conducive to antibiotic activity (6–9). C. burnetii manipulates the host cell using a type IV secretion system (T4SS) and replicates within the PV throughout a lengthy infectious cycle (5, 10–13). Currently, doxycycline effectively treats acute Q fever, which presents with flu-like symptoms and pneumonia (14, 15). However, chronic disease requires up to 1.5 years of combination therapy using doxycycline and hydroxychloroquine that often does not completely eradicate infectious bacteria (14). In addition, a doxycycline-resistant isolate of C. burnetii was recently reported (16), stressing the need for alternatives to this long-established treatment.

Host-directed compounds that prevent intracellular pathogen growth have been investigated as alternatives to traditional antibiotics (17–21), and a recent study identified 75 compounds that antagonize C. burnetii intracellular growth and typical PV expansion (22). These compounds are termed host-directed antimicrobial drugs (HDADs) because they do not directly target C. burnetii, like traditional antibiotics, but impact host processes required for intracellular growth. This study also compared the efficacy of 640 compounds against a panel of intracellular pathogens, including Brucella abortus, Rickettsia conorii, and Legionella pneumophila. The authors demonstrated multiple pathogen-specific and pan-pathogen activities of individual compounds, indicating broad applicability of HDADs in treatment of infectious diseases. In addition to drug screens, individual chemical inhibitors have been used in a similar manner to study the importance of distinct host signaling pathways during C. burnetii infection. Previous studies used chemical inhibitors to demonstrate the importance of mammalian signaling cascades, including PKA, PKC, eIF-2α, Akt, and Erk1/2, for C. burnetii replication, PV expansion, or prevention of host cell apoptosis (23–27). Together, drug screens and individual chemical inhibitor studies have demonstrated the utility of HDADs in preventing C. burnetii infection events required for host cell parasitism.

In this study, we screened an NIH clinical collection (NCC) of 727 FDA-approved or late-stage clinical trial compounds for their ability to inhibit C. burnetii growth in host cells. This screen identified 88 compounds that significantly inhibited C. burnetii growth within human THP-1 macrophage-like cells. Members of the largest group of identified inhibitory compounds target diverse components of neurotransmitter systems and have been largely used to treat psychosis and mood-related disorders. Overall, we identified a new class of anti-C. burnetii drugs that can be pursued in future studies to improve Q fever therapy.

## RESULTS

### A subset of compounds from the NCC library antagonizes C. burnetii growth in human cells.

**FIG 1:**
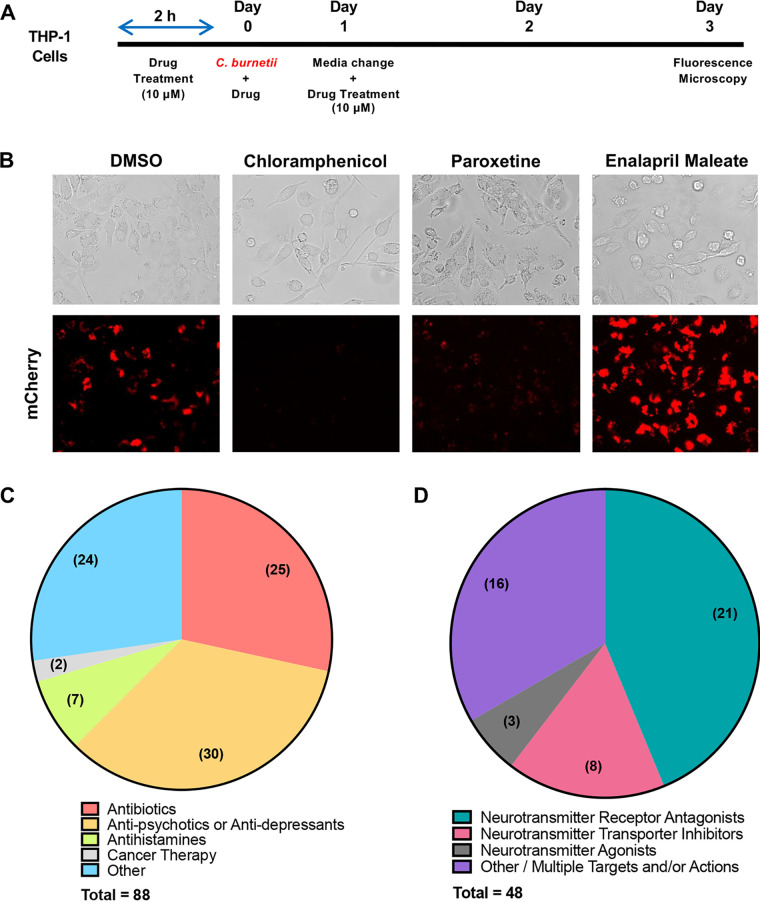
NCC compounds impact C. burnetii growth in THP-1 cells. (A) THP-1 macrophage-like cells were treated with individual NCC compounds (10 μM) or DMSO 2 h prior to infection with NMII-mCherry for 72 h. (B) Cells were processed for bright-field (top) or fluorescence (bottom) microscopy. Chloramphenicol, an antibiotic that prevents C. burnetii intracellular growth, was included as a control. Eighty-eight compounds inhibit typical C. burnetii growth (paroxetine is shown as an example). Thirty-seven compounds enhance C. burnetii growth (enalapril maleate is shown as an example). (C) Reported clinical use of the identified 88 inhibitory compounds. (D) Forty-eight inhibitory compounds target neurotransmitter system machinery. These compounds are divided into known target components and accompanying mechanism of action.

To identify new anti-C. burnetii compounds, we used the established THP-1 macrophage-like cellular infection model that is widely accepted in the C. burnetii field as an effective mimic of primary human macrophages (2, 3, 7, 9, 22–33). As shown in Fig. 1A, our initial screen consisted of differentiated THP-1 cells treated with individual drugs 2 h prior to infection with avirulent C. burnetii expressing red fluorescent mCherry (NMII-mCherry) to ensure compound effects existed at initiation of infection. At 72 h postinfection (hpi), cells were analyzed by bright-field microscopy to assess cytopathic effects (cell rounding) and fluorescence microscopy to observe intracellular accumulation of NMII-mCherry, indicative of bacterial growth. As shown in Fig. 1B and Table 1, 88 compounds prevented normal C. burnetii growth within THP-1 cells, similar to chloramphenicol treatment (34). Importantly, 52 of the 88 compounds identified have not been previously reported as anti-C. burnetii agents, indicating the discovery of novel anti-C. burnetii compounds in our screen. In contrast, 37 compounds enhanced C. burnetii replication (Fig. 1B and Table 2); however, these drugs were not pursued further in this study. The 88 inhibitory compounds were separated based on known clinical use, as shown in Fig. 1C. Unsurprisingly, 25 inhibitory compounds were common antibiotics, including levofloxacin, azithromycin, and doxycycline (the best current Q fever treatment). Seven compounds have been used as antihistamines, and the largest group with anti-C. burnetii activity included 30 antipsychotic and antidepressant drugs used to treat psychosis and mood-related disorders, respectively.

**TABLE 1:** Compounds that prevent C. burnetii replication

Compound	Reported use	Known activity	Reported^*a*^
Antibiotics			
Levofloxacin	Antibiotic	Fluoroquinolone	14, 22, 55–61
Tosufloxacin tosilate	Antibiotic	Fluoroquinolone	22
Clarithromycin	Antibiotic	Macrolide	14, 22, 62–70
Ormetoprim	Antibiotic	Folic acid synthesis inhibitor	No
Kitasamycin	Antibiotic	Macrolide	No
Rufloxacin HCl	Antibiotic	Fluoroquinolone	22
Pazufloxacin	Antibiotic	Fluoroquinolone	22
Moxifloxacin HCl	Antibiotic	Fluoroquinolone	14, 22, 56, 61, 68, 71, 72
Rifabutin	Antibiotic	Bacterial RNA synthesis inhibitor	No
Pefloxacin mesylate	Antibiotic	Fluoroquinolone	14, 22, 62, 63, 73–75
Linezolid	Antibiotic	Oxazolidinone/monoamine oxidase inhibitor	22, 63, 67
Rifaximin	Antibiotic	Bacterial RNA synthesis inhibitor	No
Enrofloxacin	Antibiotic	Fluoroquinolone	22
Eryped	Antibiotic	Macrolide	66, 76, 77
Demeclocycline	Antibiotic	Tetracycline	No
Doxycycline	Antibiotic	Tetracycline	14, 22, 55, 56, 61–63, 67, 68, 74–76, 78–87
Gatifloxacin	Antibiotic	Fluoroquinolone	22
Azithromycin	Antibiotic	Macrolide	22, 69, 88, 89
Minocycline HCl	Antibiotic	Tetracycline/5-lipoxygenase inhibitor	55, 76, 90–92
Ofloxacin	Antibiotic	Fluoroquinolone	14, 22, 57, 62, 63, 67, 74, 76, 80, 93, 94
Rifampin	Antibiotic	Bacterial RNA synthesis inhibitor	14, 22, 61, 74, 75, 80–83, 94–97
Trimethoprim^*b*^	Antibiotic	Dihydrofolate reductase inhibitor	14, 22, 84, 96–99
Chloroxine	Antibiotic	Antimitotic	No
Ethionamide	Antibiotic	Mycolic acid synthesis inhibitor	No
Oxytetracycline	Antibiotic	Tetracycline	No
Antipsychotics/Antidepressants			
Perospirone HCl	Antipsychotic	5-HT2A and D2 receptor antagonist/5-HT1A receptor partial agonist	No
Lofepramine	Antidepressant	Norepinephrine and 5-HT reuptake inhibitor/muscarinic receptor antagonist	No
Aripiprazole	Antipsychotic	Agonist, partial agonist, inverse agonist, or antagonist depending on the 5-HT or dopamine receptor(s)	22
Nefazodone	Antidepressant	5-HT1A, 5-HT2A, and α-adrenergic receptor antagonist/5-HT and norepinephrine reuptake inhibitor	No
Risperidone	Antipsychotic	5-HT, dopamine, α-adrenergic, and H1 receptor antagonist	No
Sertraline	Antidepressant	5-HT reuptake inhibitor	No
Fluphenazine 2HCl	Antipsychotic	Dopamine receptor antagonist	No
Haloperidol	Antipsychotic	D4 receptor inverse dopamine agonist/ D2 and 5-HT2A receptor antagonist	22
Fluperlapine	Antipsychotic/antidepressant	5-HT6 and 5-HT7 receptor antagonist	No
Rimcazole		σ-Receptor antagonist/dopamine reuptake inhibitor	No
Amoxapine	Antidepressant	Norepinephrine and 5-HT reuptake inhibitor/ Dopamine and 5-HT receptor antagonist	22
Chlorpromazine HCl	Antipsychotic	Dopamine, 5-HT, and H1 receptor antagonist	22
Procyclidine	Antipsychotic	Muscarinic receptor antagonist	No
Thioridazine	Antipsychotic	5-HT2A, 5-HT2C, D1, D2, α-adrenergic, and H1 receptor antagonist, cytochrome P450 2D6 inhibitor	No
Cogentin mesylate	Anti-Parkinson’s treatment	Muscarinic receptor antagonist/dopamine reuptake inhibitor	No
Paroxetine	Antidepressant	5-HT reuptake inhibitor	No
Thiothixene	Antipsychotic	Dopamine receptor antagonist	No
Duloxetine	Antidepressant	5-HT and norepinephrine reuptake inhibitor	No
Prochlorperazine	Antipsychotic	Dopamine receptor antagonist	No
Bifemelane	Antidepressant	Monoamine oxidase inhibitor	No
Indatraline		5-HT, dopamine, and norepinephrine reuptake inhibitor	No
Fluvoxamine	Antidepressant	5-HT reuptake inhibitor	No
Trifluoperazine	Antischizophrenic	Dopamine receptor antagonist	No
Maprotiline HCl	Antidepressant	Norepinephrine reuptake inhibitor	22
Perphenazine	Antipsychotic	Dopamine, H1, and α-adrenergic receptor antagonist	No
Amitriptyline HCl	Antidepressant	5-HT and norepinephrine reuptake inhibitor/5-HT2A receptor antagonist	22
Nortriptyline HCl	Antidepressant	Norepinephrine and 5-HT reuptake inhibitor/5-HT2 receptor antagonist	No
Desipramine HCl	Antidepressant	Norepinephrine and 5-HT reuptake inhibitor/5-HT2A receptor antagonist	47
Fluoxetine HCl	Antidepressant	5-HT reuptake inhibitor	22
Trihexyphenidyl	Anti-Parkinson’s treatment	Muscarinic receptor antagonist	No
Antihistamines			
Loratadine	Antihistamine	H1 receptor antagonist	22
Ketotifen fumarate	Antihistamine	H1 receptor antagonist	22
Cyproheptadine HCl	Antihistamine	5-HT, H1, and muscarinic receptor antagonist	22
Desloratadine	Antihistamine	H1 receptor antagonist	22
Phenergan (promethazine)	Antihistamine	H1 and muscarinic receptor antagonist	22
Hydroxyzine	Antihistamine	H1 receptor antagonist	No
Azelastine HCl	Antihistamine/anti-asthmatic	H1 receptor antagonist	No
Other			
Toremifene citrate	Anti-estrogen	Estrogen receptor modulator	22
Loperamide	Antidiarrheal	Opioid receptor agonist	22
Propafenone	Antiarrhythmic	Sodium and potassium channel inhibitor/calcium channel inhibitor/β-adrenergic receptor antagonist	22
RU24969 hemisuccinate		5-HT receptor agonist	No
Brucine	Anti-inflammatory/analgesic/cancer treatment	Induces apoptosis/inhibits metastasis/modulates various cell signaling pathways	No
Carvedilol	Antihypertensive	Adrenergic receptor antagonist	No
Ondansetron HCl	Antinausea	5-HT3 receptor antagonist	No
1-Benzyl imidazole	Heart-related disease treatment	α-Adrenergic receptor antagonist	No
Benproperine phosphate	Cough supplement	Inhibits afferent nerve impulses in the lungs and pleura	No
Pyrimethamine	Antimalarial/antiprotozoal	Folic acid antagonist/dihydrofolate reductase inhibitor	No
Triamterene	Diuretic/antihypertensive	Epithelial sodium channel inhibitor	No
Cisapride	Gastrointestinal agent	5-HT receptor agonist	No
Bifonazole	Antifungal	Fungal ergosterol synthesis inhibitor	22
Glimepiride	Type 2 diabetes treatment	Increases insulin secretion from β-cells	No
Vinorelbine	Cancer treatment	Antimitotic	No
Pizotyline	Migraine treatment	5-HT receptor antagonist	No
Ketoconazole	Antifungal	Fungal ergosterol synthesis inhibitor	100
S-(+)-Etomoxir	Antidiabetic	Carnitine O-palmitoyltransferase I inhibitor	No
Prednisolone acetate	Anti-inflammatory/immunosuppressive agent	Glucocorticoid receptor agonist	No
Amiodarone hydrochloride	Antiarrhythmic	Ion channel inhibitor/adrenergic receptor antagonist	22
Imatinib mesylate	Cancer treatment	Tyrosine kinase inhibitor	No
Carisoprodol	Muscle relaxant	GABA receptor modulation	No
Econazole nitrate	Antifungal	Fungal ergosterol synthesis inhibitor	No
Naphazoline	Vasoconstrictor	α-Adrenergic receptor agonist	No
Raloxifene HCl	Anti-estrogen	Estrogen receptor modulator	No
Atomoxetine HCl	Anti-ADHD	Norepinephrine reuptake inhibitor	No

a Previously reported to have anti-C. burnetii activity.

b Trimethoprim is typically used in a 1:5 mixture with sulfamethoxazole, known as cotrimoxazole. Reports cited reflect this use.

**TABLE 2:** Compounds that promote C. burnetii replication

Compound	Reported use	Known activity	Reported^*a*^
Troxipide	Gastritis cytoprotective agent	Increases mucus production and prostaglandin secretion, mucosal metabolism, and mucosal microcirculation/reduces neutrophil migration and reactive oxygen species production/regenerates collagen fibers	No
Carmofur	Cancer treatment	Pyrimidine analog that inhibits thymidylate synthase	No
4-Chloro-N-(2-morpholin-4-yl-ethyl)-benzamide	Antidepressant	Monoamine oxidase inhibitor	No
Triclabendazole	Antihelminthic	Helminth motility inhibitor leading to parasite death	No
Tacrolimus	Immunosuppressive agent	Reduces cytokine production/T-cell activation inhibitor	No
Stavudine	Anti-HIV	Nucleoside reverse transcriptase inhibitor	No
Ftorafur	Cancer treatment	Thymidylate synthase inhibitor/interrupts RNA functions	No
Hyperoside	Flavonoid		No
Ticlopidine HCl	Anti-platelet	Adenosine diphosphate receptor inhibitor	No
Rizatriptan benzoate	Migraine treatment	5-HT1 receptor agonist	No
Nicorandil	Anti-anginal	Opens ATP-dependent potassium channels to induce vasodilation	No
MK-886	Cancer treatment	5-Lipoxygenase inhibitor/leukotriene antagonist	No
Indirubin	Cancer or psoriasis treatment	CDK inhibitor/GSK3-β inhibitor/induces apoptosis	No
Azasetron	Antinausea	5-HT3 receptor antagonist	No
Diphenoxylate	Antidiarrheal	Opioid receptor agonist	No
Vinorelbine tartrate	Cancer treatment	Tubulin polymerization inhibitor	No
Huperzine A	Swelling, fever, and blood disorder treatment/Alzheimer’s disease treatment/myasthenia gravis treatment	NMDA receptor antagonist/reversible acetylcholinesterase inhibitor	No
CCPA			No
Zaleplon	Insomnia treatment	GABA-A receptor agonist/GABA-BZ receptor modulator	No
Hydrocortisone hemisuccinate	Anti-inflammatory (dermatitis treatment)/immunosuppressive agent	Glucocorticoid receptor agonist	No
Indapamide	Antihypertensive/diuretic	Inhibits K^+^ flow through ion channels and uptake of Na^+^ and Cl^−^ ions	No
Labetalol HCl	Antihypertensive	Adrenergic receptor antagonist	No
Medrysone	Anti-inflammatory (eye inflammation treatment)	Glucocorticoid receptor agonist	No
Selegiline HCl	Anti-Parkinson’s treatment	Monoamine oxidase inhibitor	No
Nafcillin	Antibiotic	β-Lactam	No
Beclomethasone dipropionate	Anti-asthmatic/anti-inflammatory	Glucocorticoid receptor agonist	No
Griseofulvin	Antifungal	Fungal microtubule interference	No
Enalapril maleate	Antihypertensive	ACE inhibitor	No
Naltrexone HCl	Opiod addiction and alcohol dependence treatment	Opioid receptor antagonist	No
Simvastatin	High cholesterol treatment	HMG-CoA reductase inhibitor	No
Deferiprone	Thalassemia treatment	Iron chelator	No
Diclofenac sodium salt	Nonsteroidal anti-inflammatory	COX inhibitor	No
Zucapsaicin	Osteoarthritis treatment	TRPV-1 modulator	No
Nornicotine	Smoking cessation treatment	Nicotine receptor agonist	No
Lidocaine	Anesthetic/antiarrhythmic	Sodium channel inhibitor	No
Indomethacin	Nonsteroidal anti-inflammatory	COX inhibitor	No
LY171883 (tomelukast)	Anti-asthmatic	Leukotriene agonist	No

a Drugs previously reported to impact C. burnetii growth.

Antipsychotic and antidepressant drugs often target distinct neurotransmitter machinery components. Eighteen identified inhibitory compounds outside the antipsychotic and antidepressant group also target neurotransmitter machinery. These results make neurotransmitter systems the target of over half of the inhibitory compounds (48 of 88) identified in this screen. Figure 1D separates these compounds by known target and mechanism of action. The largest subset of this group antagonizes neurotransmitter receptors, and members of the smallest subset act as neurotransmitter agonists. This category of compounds has not been previously explored for anti-C. burnetii properties and represents a novel class of potential HDADs that antagonize C. burnetii replication.

### Neurotransmitter system-targeting compounds prevent typical C. burnetii intracellular growth.

To further investigate neurotransmitter system-targeting compounds as HDADs against C. burnetii, we assessed a representative sampling of these drugs (28 of the 48 compounds identified in Fig. 1). Most of these compounds are antipsychotic or antidepressant drugs and represent the largest HDAD group, targeting diverse neurotransmitter system components. In growth inhibitor screens, it is critical to differentiate compounds that specifically inhibit bacterial growth within host cells from those that are directly toxic to bacteria using a traditional antibiotic mode of action. To distinguish between these two scenarios, we compared C. burnetii treated with individual compounds during infection of THP-1 cells to treated bacteria growing in axenic media. It is also important to differentiate between compounds that prevent bacterial entry into host cells and those that prevent intracellular growth following uptake, which mimics treatment of a previously infected patient. Thus, all compounds were added at 24 hpi to ensure that growth defects resulted from inhibiting intracellular growth independent of host cell uptake.

Of the 28 inhibitory neurotransmitter system-targeting compounds identified, only aripiprazole and nefazodone were substantially cytotoxic to THP-1 cells (Table S1), with no direct antibacterial effects on C. burnetii (Table 3). Lofepramine was the only compound that reduced C. burnetii growth in axenic media by almost 50% compared to vehicle control-treated cultures at 7 days postinoculation, suggesting the drug acts similar to a traditional antibiotic on C. burnetii at the concentration tested. Atomoxetine was the only compound that demonstrated no detectable inhibitory effect on C. burnetii intracellular growth (Table 4) or in axenic media. These data, combined with the observation that pretreatment with atomoxetine inhibits C. burnetii growth (Fig. 1A and Table 1), suggests the drug prevents bacterial entry into host cells. Amoxapine and perospirone treatment resulted in less than 30% reduction in intracellular growth compared to vehicle control-treated cells at 5 days postinfection (dpi). The former had no direct antibacterial effect, while the latter reduced C. burnetii growth by less than 20% compared to vehicle control-treated axenic cultures. This result suggests inhibitory effects observed in our original microscopy screen (Fig. 1A) are due to reduced bacterial entry. Of the remaining 22 identified compounds, 18 reduced intracellular growth by more than 45% and 4 reduced intracellular growth by more than 30%. While 11 of these 22 compounds had statistically significant antibacterial effects, none reduced C. burnetii growth in axenic media more than 30% at 7 days postinoculation. Collectively, we identified only one potential direct antibacterial compound and 22 neurotransmitter machinery-targeting HDADs that prevent typical intracellular C. burnetii growth.

**TABLE 3:** Effect of neurotransmitter system-targeting compounds on C. burnetii axenic growth

Compound	C. burnetii axenic growth^*a*^ (%)			
	5 days	SD	7 days	SD
Antibacterial candidate				
Lofepramine	79.28	36.32	56.73****	9.25
Bacterial entry prevention candidates				
Atomoxetine HCl	93.33	29.41	91.63	17.85
Amoxapine	114.1	32.07	92.96	17.49
Perospirone HCl	83.25	24.66	81.91*	22.95
Identified HDADs				
Amitriptyline HCl	102	23.58	91.33	20.22
Bifemelane HCl	98.28	43.79	81.34*	22.84
Chlorpromazine HCl	99.22	34.54	96.53	18.83
Cisapride monohydrate	76.04**	23.39	84.22**	18.25
Cogentin Mesylate	104.9	47.55	91.69	18.27
(*S*)-Duloxetine HCl	85.96	22.73	81.35	21.15
Fluphenazine 2HCl	86.3	37.57	76.59**	23.84
Fluvoxamine maleate	94	18.9	103.1	16.68
Haloperidol	98.37	38.36	95.71	16.11
Indatraline HCl	93.04	32.49	76.01*	19.42
Maprotiline HCl	96.51	29.96	100.8	14.65
Nortriptyline HCl	94.75	24.03	103.4	24.26
Paroxetine maleate salt	102.9	67.23	99.48	20.58
Perphenazine	125.6	68.27	102.7	26.09
Prochlorperazine dimaleate salt	119	68.02	85.98*	12.72
Procyclidine HCl	85.6	22.88	78.68**	20.9
Rimcazole 2HCl	72.68**	20.1	75.16**	19.34
Risperidone	78.56*	18.02	71.39****	10.62
Sertraline HCl	119.9	75.44	92.88	22.2
Thioridazine HCl	81.59	32.97	78.68**	19.34
(Z)-Thiothixene	96.45	20.66	96.59	24.45
Trifluoperazine 2HCl	77.17*	17.37	78.86*	24.5
Cytotoxic to THP-1 cells				
Aripiprazole	107.7	48.96	90.38	25.1
Nefazodone HCl	106.3	20.79	99.96	13.88

a Student’s *t* test was used to compare percent growth of C. burnetii in compound-treated cultures to DMSO-treated cultures. SD, standard deviation from the mean. *, *P *< 0.05; **, *P *< 0.01; ****, *P *< 0.0001.

**TABLE 4:** Effect of neurotransmitter system-targeting compounds on C. burnetii intracellular growth

Compound	C. burnetii growth (%) in THP-1 cells	SD^*a*^
Antibacterial candidate		
Lofepramine	65.76****	19.95
Prevent bacterial entry candidates		
Atomoxetine HCl	94.19	19.85
Amoxapine	70.26****	12.81
Perospirone HCl	77.18**	26.49
Identified HDADs		
Amitriptyline HCl	66.81****	10.51
Bifemelane HCl	41.86****	13.40
Chlorpromazine HCl	55.53****	13.9
Cisapride monohydrate	45.53****	21.53
Cogentin mesylate	47.37****	12.17
(*S*)-Duloxetine HCl	28.54****	11.48
Fluphenazine 2HCl	37.34****	9.18
Fluvoxamine maleate	49.34****	10.13
Haloperidol	65.84****	19.56
Indatraline HCl	22.73****	9.23
Maprotiline HCl	44.81****	13.79
Nortriptyline HCl	38.08****	14.32
Paroxetine maleate salt	31.82****	14.31
Perphenazine	31.26****	10.69
Prochlorperazine dimaleate salt	28.64****	14.05
Procyclidine HCl	66.49****	13.3
Rimcazole 2HCl	18.47****	15.76
Risperidone	46.78****	21.99
Sertraline HCl	15.27****	11.1
Thioridazine HCl	29.98****	7.73
(Z)-Thiothixene	20.11****	10.68
Trifluoperazine 2HCl	32.26****	12.96

a Student’s *t* test was used to compare percent growth of C. burnetii in compound-treated infections to DMSO-treated infections. SD, standard deviation from the mean. **, *P *< 0.01; ***, *P* < 0.001; ****, *P *< 0.0001.

10.1128/mSphere.00442-21.1 TABLE S1 Cytotoxicity of neurotransmitter system-targeting compounds. Download Table S1, DOCX file, 0.01 MB. Copyright © 2021 Fullerton et al. 2021 Fullerton et al. https://creativecommons.org/licenses/by/4.0/ This content is distributed under the terms of the Creative Commons Attribution 4.0 International license.

**FIG 2:**
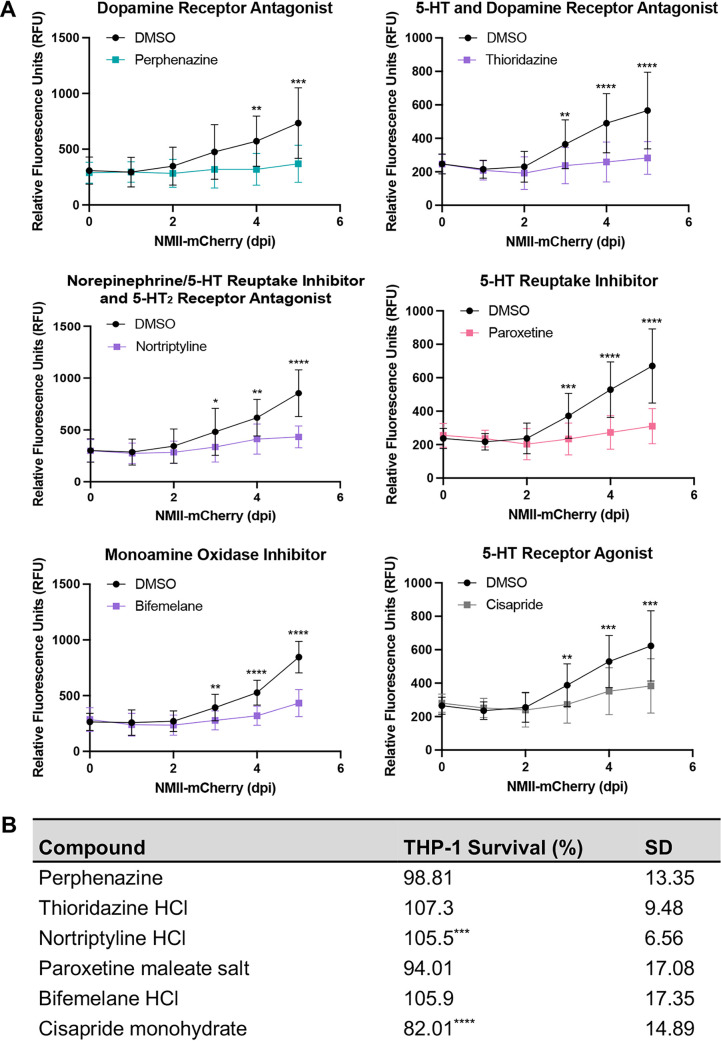
HDADs targeting host monoamine neurotransmitter systems inhibit C. burnetii growth in THP-1 cells. (A) THP-1 cells infected with NMII-mCherry were treated with DMSO or the indicated compounds (10 μM) at 24 hpi. mCherry fluorescence was measured for 5 days as a readout of bacterial replication. Error bars represent standard deviations (SD) from the means. *, *P < *0.05; **, *P < *0.01; ***, *P < *0.001; ****, *P < *0.0001. (B) Macrophage survival was assessed at 5 dpi following the infection and treatment scheme in panel A. Uninfected, untreated cells served as a control for survival, while uninfected cells treated with DMSO (10%) for 24 h served as a cell death control. Compound-treated, infected cells were compared to DMSO-treated, infected cells. ***, *P < *0.001; ****, *P < *0.0001. Each compound shown significantly suppresses C. burnetii intracellular growth without causing substantial THP-1 cell death.

Multiple neurotransmitter systems and components are targeted by the identified HDADs. Figure 2A shows fluorescence-based 5-day NMII-mCherry growth curves in THP-1 cells treated with representative compounds that target diverse components of monoamine neurotransmitter systems, including the serotonin (also known as 5-hydroxytryptamine or 5-HT), dopamine, or norepinephrine systems. Perphenazine and thioridazine are antipsychotic compounds that antagonize dopamine receptors or 5-HT and dopamine receptors, respectively. Nortriptyline, paroxetine, and bifemelane are antidepressant compounds. Nortriptyline and paroxetine target monoamine transporters, inhibiting reuptake of extracellular norepinephrine and/or 5-HT into host cells. Nortriptyline also antagonizes 5-HT_2_ receptors. Bifemelane inhibits monoamine oxidases involved in monoamine metabolism. In contrast, cisapride is a 5-HT agonist that has been used to treat gastrointestinal ailments. All 6 example compounds inhibited typical C. burnetii intracellular replication from 3 to 5 dpi. Importantly, inhibitory effects were not due to THP-1 cell cytotoxicity (Fig. 2B) or direct bactericidal effects (Table 3). Overall, our results suggest diverse components of monoamine neurotransmitter systems can be targeted to inhibit C. burnetii intracellular growth.

### HDADs prevent expansion of the C. burnetii replication vacuole.

**FIG 3:**
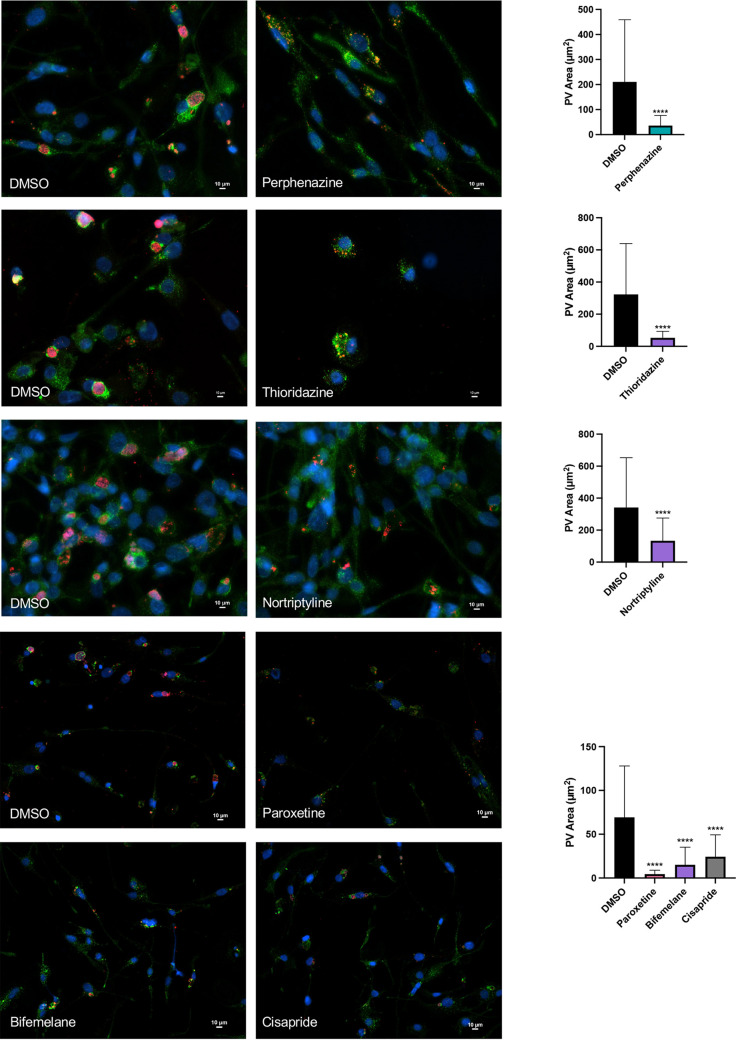
HDADs targeting host monoamine neurotransmitter systems inhibit C. burnetii PV expansion. NMII C. burnetii-infected THP-1 cells were treated with DMSO or the indicated compounds (10 μM) at 24 hpi and processed for fluorescence microscopy at 96 hpi. Antibodies were used to detect C. burnetii (red) and CD63 (late phagosomal PV marker; green). DNA was stained with DAPI (blue). The area of 50 PV was measured under each condition. Error bars represent the standard deviations from the means. ****, *P* < 0.0001. Each HDAD shown prevents normal PV expansion.

To replicate within eukaryotic cells, C. burnetii must form a phagolysosome-like PV to activate metabolism and allow bacterial cell division as the vacuole expands (35–37). To determine if HDADs that target monoamine neurotransmitter system components disrupt PV expansion, we measured PV area in infected THP-1 cells treated with perphenazine, thioridazine, nortriptyline, paroxetine, bifemelane, or cisparide using fluorescence microscopy. As shown in Fig. 3, each compound prevented typical PV expansion when added to cells at 24 hpi, indicating PVs were unable to undergo heterotypic fusion and appropriately expand. These results suggest that host 5-HT, dopamine, or norepinephrine system activity positively impacts C. burnetii PV expansion needed for bacterial replication to high numbers.

### HDADs antagonize virulent C. burnetii replication in primary human alveolar macrophages.

**FIG 4:**
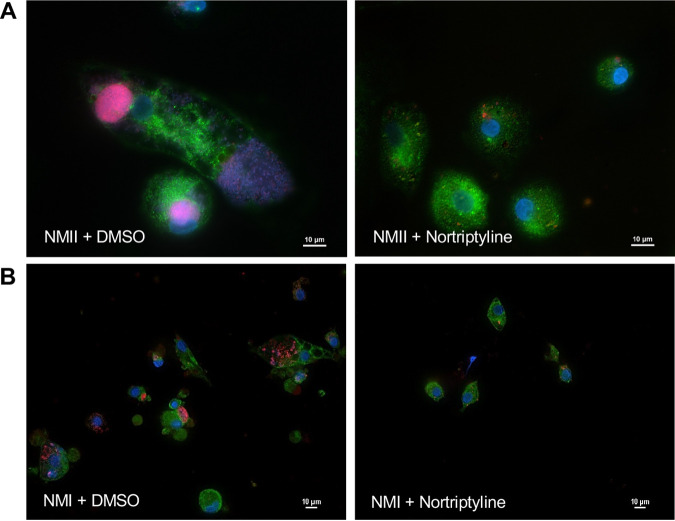
Nortriptyline reduces virulent C. burnetii growth and PV expansion in primary hAMs. Primary hAMs were infected with NMII-mCherry (A) or virulent NMI C. burnetii (B) and treated with DMSO (left) or nortriptyline (10 μM; right) at 24 hpi. Cells were processed for fluorescence microscopy at 96 hpi. mCherry fluorescence (top; red) or anti-C. burnetii antibody (bottom; red) indicates bacteria, antibody against CD63 (green) is labeled the PV, and DNA was stained with DAPI (blue). Inhibitors prevent typical PV expansion by avirulent and virulent C. burnetii in primary hAMs.

THP-1 cells have been used in numerous studies as a reliable *in vitro* model of C. burnetii interactions with human macrophages (7, 27, 28). However, cell line results should be confirmed in primary cells when possible to ensure disease relevance, particularly when assessing new HDADs. We previously established primary human alveolar macrophages (hAMs) as a disease-relevant system to determine if *in vitro* findings extend to cells preferentially targeted by C. burnetii in the human lung (3, 29, 38). Here, nortriptyline was used as a representative monoamine neurotransmitter system-targeting, anti-C. burnetii HDAD. hAMs were infected with avirulent NMII-mCherry and treated with nortriptyline at 24 hpi. Reduced NMII-mCherry within hAMs (Fig. 4A) confirmed that the inhibitory properties of this compound, which correlates with findings from our THP-1 cell line model, are reproducible in a disease-relevant context. Next, we treated hAMs infected with virulent NMI C. burnetii (acute disease isolate) with nortriptyline at 24 hpi and monitored PV expansion. Nortriptyline efficiently prevented PV expansion and accumulation of large numbers of virulent C. burnetii in hAMs (Fig. 4B). Together, these results indicate targeting host monoamine neurotransmitter system machinery is a therapeutic strategy relevant in a natural disease cellular setting involving virulent C. burnetii.

## DISCUSSION

In this study, we identified 88 compounds with anti-C. burnetii activity and validated 22 of these drugs as potential HDADs to treat Q fever. New therapies are desperately needed to combat C. burnetii infection, particularly for chronic disease, the most life-threatening form of Q fever. The current regimen for treating Q fever endocarditis is up to 1.5 years of doxycycline treatment combined with a pH-elevating compound (14). This time course is inefficient, and treatment does not always clear infectious bacteria. As an alternative to traditional antibiotics, multiple studies have repurposed host-directed compounds that target eukaryotic proteins usurped by intracellular pathogens (17–19, 22). A key feature of these HDADs is the ability to suppress bacterial replication or degrade bacteria while maintaining host cell viability despite altering host processes. Therapeutics of this nature hold great promise in the current era of antibiotic resistance. HDADs are problematic for disease-causing bacteria because they do not target the pathogen directly, making development of classical genetics-based resistance unlikely and ultimately improving treatment.

Based on our extensive drug screen, 22 identified compounds specifically inhibit C. burnetii intracellular growth in a host-directed manner. Our *in vitro* experimental approach mimics a natural scenario in which a patient receives treatment after infection and diagnosis. The majority of these compounds have been used extensively to treat a variety of psychoses or mood disorders, including schizophrenia and depression, respectively. These drugs target distinct components of neurotransmitter systems, most commonly the serotoninergic, dopaminergic, and adrenergic systems comprised of components that respond to 5-HT, dopamine, or epinephrine and norepinephrine. Prior to this study, Czyż et al. assessed the antibacterial activity of 640 compounds against L. pneumophila, B. abortus, R. conorii, and C. burnetii, identifying 75 HDADs that prevent typical C. burnetii growth (22). Many of these compounds target G protein-coupled receptors (GPCRs), intracellular calcium signaling, or sterol homeostasis machinery. In line with these findings, we found many compounds that significantly impede C. burnetii intracellular growth and target neurotransmitter receptors that are part of the GPCR family. Although the parameters between studies were not identical, Czyż et al. reported 4 of the 22 HDADs identified in our study, confirming the effectiveness of our approach in replicating previously confirmed HDADs. Of these compounds, 2 also antagonized growth of L. pneumophila, suggesting broader applicability in infectious disease treatment. Compounds assessed by Czyż et al. and many of the compounds in the current study have already been approved by the FDA to treat disorders unrelated to infection, suggesting they can be safely administered to humans, but they have not been used to treat Q fever.

Neurotransmitter system-targeting drugs also alter the life cycle of other intracellular pathogens beyond those reported by Czyż et al. and our current study. For example, Mycobacterium tuberculosis is sensitive to thioridazine and nortriptyline, two compounds with anti-C. burnetii activity in our study, further supporting the potential for broad-spectrum use (39–41). In addition, pimozide, a dopamine receptor inhibitor used to treat schizophrenia and Tourette’s syndrome, reduces entry of Listeria monocytogenes into host phagocytes and host entry and intracellular replication of the eukaryotic parasite Toxoplasma gondii (17, 18). Interestingly, the effect of pimozide on T. gondii replication is independent of neurotransmitter receptor signaling. As demonstrated for T. gondii, it is important to note the potential for off-target effects when conducting drug studies. Therefore, while neurotransmitter system-targeting compounds represent a major class of potential therapeutics to treat intracellular pathogen infections, future studies should investigate whether anti-C. burnetii activity is due to traditional or alternative activities of these compounds.

At the whole-host level, neurotransmitter systems are not typically considered in mechanistic studies of respiratory infections. However, numerous reports demonstrate a role for 5-HT, dopamine, and norepinephrine in macrophage function that could impact alveolar physiology. For example, 5-HT impacts alveolar macrophage production of tumor necrosis factor alpha (TNF-α) and interleukin-10 (IL-10) (42), which are involved in proinflammatory and anti-inflammatory responses, respectively. In addition, norepinephrine signaling through β_2_-adrenergic receptors may drive macrophage IL-10 production (43). C. burnetii triggers a robust human macrophage inflammatory response characterized by production of TNF-α, IL-6, and IL-8, and the pathogen stimulates anti-inflammatory IL-10 production (3, 29). Thus, HDAD alteration of the innate immune response may promote C. burnetii clearance and less severe acute disease. In line with this prediction, dopamine receptor activity modulates production of IL-6 and TNF-α (44), and 5-HT signaling impacts production of the monocyte chemoattractant CCL2 (45). Other bacterial pathogens are similarly susceptible to HDADs that alter the inflammatory response. For example, resolvin and clavanin modulate immune responses to Escherichia coli and Staphylococcus aureus, indicating that dampening the host inflammatory response prevents robust bacterial growth, effectively inhibiting disease progression (46–48). Moreover, neurotransmitter receptor expression and signaling impacts macrophage polarization that defines the inflammatory state of the cell (43, 49). C. burnetii promotes alveolar macrophage transition from M1 to M2 polarization to provide a more hospitable growth niche (3), and 5-HT, dopamine, or norepinephrine signaling may contribute to this event. Thus, future studies should determine if neurotransmitter signaling controls cytokine/chemokine production by, and polarization of, C. burnetii-infected macrophages.

At the cellular level, some neurotransmitter system-targeting compounds that inhibit PV expansion impact host autophagy. Autophagy is a homeostatic process that recycles damaged cytosolic components, regulates inflammation, and clears invading bacteria by delivery to degradative lysosomes (50, 51). However, many intracellular pathogens modulate this process for their own benefit, including C. burnetii. PV expansion involves T4SS-dependent recruitment of, and fusion with, autophagosomes (1, 7, 52, 53). Multiple antipsychotic and antidepressant drugs, including thioridazine, nortriptyline, and paroxetine, can induce autophagy by increasing processing of autophagy protein microtubule-associated light chain 3 (LC3) or modulating mammalian target of rapamycin (mTOR), a kinase component of the autophagy regulator mTOR complex 1 (mTORC1) (39, 50, 51). During infection, LC3 is recruited to the PV, and *Coxiella* vacuolar protein F (CvpF) promotes LC3 processing to its lipidated form (LC3-II), demonstrating that autophagy is manipulated by the pathogen (1, 7, 52, 53). C. burnetii also inhibits mTORC1 in a T4SS-dependent manner to promote PV expansion and bacterial replication (2). As with any homeostatic process, activation and deactivation of autophagy is a delicate balance that can favor the pathogen or host depending on their respective needs. Thus, although C. burnetii actively recruits autophagosomes, HDADs may overactivate autophagy, or reroute autophagic machinery, to negate proper PV expansion. Together, these findings provide a basis for future mechanistic studies of HDAD prevention of C. burnetii intracellular growth.

Overall, our drug screen results present multiple new options for future anti-Q fever therapeutic investigation. These options are needed in light of the nonspecific flu-like nature of acute disease and the potential for chronic infection leading to life-threatening endocarditis. The immunomodulatory role of peripheral neurotransmitters on macrophages suggests the traditional activity of neurotransmitter system-targeting compounds should not be dismissed when investigating anti-C. burnetii activity. These potential therapeutics now await testing in animal models to assess utility as anti-Q fever treatments. This testing will ultimately provide novel therapies that suppress disease progression following C. burnetii infection and limit pathogen development of resistance due to host-directed activity. Moreover, due to the wide-ranging effect of specific antipsychotic or antidepressant drugs on multiple intracellular pathogens, these therapies may serve broad-spectrum purposes in infectious disease treatment. For example, a study by Cao et al. used the compound library in the current study to identify drugs that prevent mouse hepatitis virus infection (54). Although addressing potential side effects of neurotransmitter system-targeting drugs would be critical prior to human administration, these HDADs hold immense promise as antimicrobial agents that potentially can be used to combat multiple, disparate infections.

## MATERIALS AND METHODS

### Bacterial and eukaryotic cell culture.

Avirulent (Nine Mile Phase II [NMII]; RSA 439) Coxiella burnetii expressing fluorescent mCherry (NMII-mCherry) was cultured in acidified citrate cysteine medium 1 (ACCM-1) containing chloramphenicol (3 μg/ml) at 37ºC, 5% CO_2_, and 2.5% O_2_. After 7 days, bacterial cultures were pelleted by centrifugation and washed with 250 mM sucrose phosphate (SP) buffer. Bacterial stocks were stored in SP buffer at −80ºC. Wild-type NMII or virulent C. burnetii (Nine Mile I [NMI]; RSA 493) isolates were cultured, harvested, and stored as described above without antibiotic. A multiplicity of infection of 10 to 30 was used for each experiment. Experiments using virulent C. burnetii were conducted in the UAMS biosafety level-3 laboratory approved by the Centers for Disease Control and Prevention.

THP-1 cells (TIB-202; American Type Culture Collection) were cultured in RPMI 1640 medium (Gibco) containing 10% fetal bovine serum (FBS; Bio-techne) at 37ºC and 5% CO_2_. Before infection, THP-1 cells were differentiated into macrophage-like cells by incubating with medium containing phorbol 12-myristate 13-acetate (PMA; 200 nM; Calbiochem) overnight. PMA-containing medium was removed prior to infection.

Primary human alveolar macrophages (hAMs) were isolated from human lungs postmortem (National Disease Research Interchange) by bronchoalveolar lavage (BAL) as previously described (29). BAL fluid was centrifuged and 0.86% ammonium chloride added to lyse red blood cells. Dulbecco’s modified Eagle’s medium/F-12 (DMEM/F-12; Gibco) containing 10% FBS, 1% antibiotic-antimycotic (10,000 U/ml penicillin, 10,000 μg/ml streptomycin, and 25 μg/ml amphotericin B; Gibco), and gentamicin (10 μg/ml; Gibco) was used to neutralize the lysis reaction. Cells were allowed to adhere to tissue culture dishes for 1.5 to 2 h at 37ºC and 5% CO_2_. Culture medium was then replaced with fresh medium to remove nonadherent cells. Medium was replaced every other day for 1 week, and at least 24 h prior to infection, medium was replaced with antibiotic-antimycotic-free media.

### Small-molecule screen.

THP-1 cells cultured on glass coverslips in 24-well plates were treated with individual compounds (10 μM) or dimethyl sulfoxide (DMSO) 2 h prior to infection with NMII-mCherry. Compounds were obtained from the NIH Clinical Compound Library (NIH Clinical Collection 1 and 2). At 24 h postinfection (hpi), medium was replaced with fresh media containing fresh compounds. Cells were fixed with ice-cold methanol and blocked with PBS containing 0.5% bovine serum albumin (BSA; Cell Signaling) at 72 hpi. Coverslips were mounted onto slides with MOWIOL (Sigma-Aldrich). Bright-field microscopy was used to visualize infected cells, and mCherry expression allowed visualization of C. burnetii (Nikon Ti-U microscope).

### Cytotoxicity assay.

THP-1 cells cultured in 96-well clear, flat-bottom plates were infected with NMII-mCherry, and the inoculum was removed and replaced with fresh medium containing DMSO or individual drugs (10 μM) at 24 hpi. Medium in cell death control wells was replaced with medium containing DMSO (10%) 24 h prior to the endpoint. A Cell Counting Kit-8 (Dojindo Laboratories) was used according to the manufacturer’s instructions at 5 days postinfection (dpi) to detect viable cells and calculate percent survival.

### Intracellular bacterial growth assay.

THP-1 cells were cultured in RPMI 1640 phenol red-free medium (Gibco) supplemented with 10% FBS in 96-well glass, flat-bottom black plates. Cells were infected with NMII-mCherry and then treated with medium containing DMSO or individual drugs (10 μM) following removal of the inoculum at 24 hpi. mCherry fluorescence was measured for 5 days, starting at day 0, using a Biotek Synergy H1 microplate reader (excitation at 585 nm and emission at 620 nm [Ex_585_/Em_620_]). Bacterial growth was calculated using the formula percent growth = [(sample − average uninfected)/(average DMSO − average uninfected)] × 100.

### Axenic bacterial growth assay.

NMII-mCherry was grown in 96-well glass, flat-bottom, black plates in ACCM-1 treated with DMSO or individual drugs (10 μM). Starting at day 0, mCherry fluorescence was measured for 7 days with a Biotek Synergy H1 microplate reader (Ex_585_/Em_620_). Cultures were mixed every other day. Bacterial growth was calculated using the formula percent growth = [(sample − average ACCM-1)/(average DMSO − average ACCM-1)] × 100.

### Immunofluorescence microscopy.

THP-1 cells or primary hAMs, plated on glass coverslips in a 24-well plate, were infected with NMII-mCherry, wild-type NMII, or virulent NMI C. burnetii. The inoculum was removed and replaced with fresh medium containing DMSO or individual drugs (10 μM) at 24 hpi. At 96 hpi, cells were washed with cold PBS three times and fixed with PBS containing 4% formaldehyde for 15 min or ice cold methanol for 3 to 5 min. Cells were washed with cold PBS and blocked overnight at 4°C in PBS containing 0.5% BSA (methanol-fixed) or this solution supplemented with 0.3% Triton X-100 (formaldehyde-fixed cells). Cells were then incubated with the appropriate block solution containing primary antibodies for 1 h with rocking at room temperature (RT). Cells were then washed in cold PBS and placed in the appropriate block solution containing secondary antibodies for 1 h with rocking at RT. Cells were washed with cold PBS and then incubated with 4′,6-diamidino-2-phenylindole (DAPI; Invitrogen) for 5 min. Coverslips were mounted onto slides with MOWIOL. Images were acquired using a 40× objective or under oil immersion using a 60× objective with either a Nikon Ti-U Eclipse One microscope or Nikon Ti2 Eclipse microscope. A D5-QilMc digital camera was used to obtain images shown in Fig. 3 (perphenazine, thioridazine, and nortriptyline) and Fig. 4A, and a DS-Qi2 digital camera was used to acquire images shown in Fig. 3 (paroxetine, bifemelane, and cisapride) and Fig. 4B. NIS elements software (Nikon) was used to measure the areas (NMII-infected THP-1 cells) or diameter (NMI-infected hAMs) of 50 PV, which were then averaged. Primary antibodies were used to detect CD63 (BD Biosciences) or C. burnetii. Secondary antibodies used were mouse antibody conjugated to Alexa Fluor 488 and rabbit or guinea pig antibodies conjugated to Alexa Fluor 594.

### Statistical analysis.

All statistical analyses were performed using Student's *t* test and Prism software (GraphPad 8 or 9). A *P* value of <0.05 was considered significant in all experiments.
