# Supplementary material for: Neurotransmitter System-Targeting Drugs Antagonize Growth of the Q Fever Agent, Coxiella burnetii, in Human Cells
Source: mSphere. 2021 Jul 7;6(4):e00442-21. doi: 10.1128/mSphere.00442-21 (PMC8386451; doi:10.1128/mSphere.00442-21)
Supplement: TABLE S1 [file msphere.00442-21-st001.docx]

**TABLE S1**

Cytotoxicity of neurotransmitter system-targeting compounds

| **Compound** | **THP-1 Survival (%)** | **SD^a^** |
| --- | --- | --- |
| Amitriptyline HCl | 99.38 | 6.26 |
| Amoxapine | 104.6^*^ | 8.27 |
| Aripiprazole | 57.17^****^ | 26.49 |
| Atomoxetine HCl | 104.7 | 7.19 |
| Chlorpromazine HCl | 115.1 | 12.73 |
| Cogentin Mesylate | 108 | 12.12 |
| (S)-Duloxetine HCl | 96.19 | 4.462 |
| Fluphenazine 2HCl | 111.4 | 11.91 |
| Fluvoxamine maleate | 117.7 | 8.32 |
| Haloperidol | 100.8 | 9.26 |
| Indatraline HCl | 91.53 | 6.65 |
| Lofepramine | 107 | 12.42 |
| Maprotiline HCl | 103.2^*^ | 8.21 |
| Nefazodone HCl | 62.27^****^ | 14.99 |
| Perospirone HCl | 81.16^***^ | 25.79 |
| Prochlorperazine dimaleate salt | 107 | 16.69 |
| Procyclidine HCl | 109 | 7.81 |
| Rimcazole 2HCl | 105.1 | 17.34 |
| Risperidone | 77.58^***^ | 16.96 |
| Sertraline HCl | 101.5 | 15.51 |
| (Z)-Thiothixene | 98.97 | 15.37 |
| Trifluoperazine 2HCl | 93.1^**^ | 21.41 |

^a^Student’s T test used to compare THP-1 survival in compound-treated *C. burnetii* infections to DMSO-treated infections. SD = standard deviation from the mean. *, *P* < 0.05; **, *P* < 0.01; ***, P < 0.001; ****, *P* < 0.0001.
